# Broiler genetics and the future outlook

**DOI:** 10.3389/fphys.2023.1150620

**Published:** 2023-03-08

**Authors:** Paul B. Siegel

**Affiliations:** School of Animal Sciences, Virginia Tech, Blacksburg, VA, United States

**Keywords:** broiler, chicken, genetics, breeding, history

While preparing this essay, the quote by Abner Kovner kept recurring.

“To Remember the Past.

To Live the Present.

To Trust the Future”

Hanging on the wall of my study is a plaque, dated 1948, that reads “The Chicken-of-Tomorrow Committee presents this Certificate of Quality to Paul Siegel for outstanding achievement in breeding and development of superior meat-type chickens.” That was 75 years ago, and it is only during the past 100 years that the production of chickens for human meat consumption was no longer a by-product of the commercial egg industry. This comment may be surprising unless we recognize that the domestication of the chicken from its wild ancestry is recent in the context of human history (Smith and Daniel, 1975). Moreover, among domesticated farm animals, the chicken increased in size while mammals became smaller (Diamond, 1995). In an evolutionary context, the domestication of the chicken had not been great, as Jungle Fowl cross fully with domestic chickens (Sutherland et al., 2018).

There is a wealth of literature on the domestication of the chicken for religious, cultural, and sport reasons. Its origins and roles as a food source too was beautifully discussed essentially a century ago in the National Geographic magazine (Jull, 1927, 1930; Lewis, 1927). For broilers, examples of anthologies include Gordy (1974), Watts (1996), Cahaner and Siegel (1986), and Siegel (2014, 2018). These publications and others reveal that it is only during the past 100 years that the broiler ceased to be a by-product of the commercial egg industry, fostered by Cecile Steele, with a subsequent focus on meat (broilers and broiler genetics).

Initially, the process of producing broilers *via* broiler genetics involved the development of the brown egg “dual purpose” chicken. Males were still reared for meat and females for egg production. It was post World War II when the “Chicken-of-Tomorrow” program (Gordy, 1974) provided the impetus for the development of breeding programs explicitly genetically designed for the production of a commercial meat-type (broiler) chicken. The initial stocks, which consisted of line crosses, were distinct from that of dual purpose chickens. Thus, although the chicken was domesticated during Neolithic times, the development of genetic programs designed for broiler performance (meat) was a 20th century event. The rapid development of a broiler *per se* was based on available stocks and sound breeding principles based on development of qualitative and quantitative genetics, which were first demonstrated in animals early in the first decade of the 20th century [e.g., Bateson and Punnett, 1959 (1905–1908)]. Broiler genetics, although conceptually new, was founded on a solid biological background.

The plethora of literature on the reduction in time and feed required to produce today’s “broiler” is a story well documented and beyond the scope of this essay. Yet, it is instructive to review the numerous traits that favored domestication of the chicken. Although some of these are no longer relevant in current broiler production due to human intervention, they are necessary to our understanding of why domestication of the chicken was not complicated. They were small and did not migrate, there were social groupings of males and females, and they possessed behavior traits such as promiscuity and broodiness. Precocial young, with well-developed motor ability and auditory and tactile senses, contributed to an adaptation to a range of environments (Hale, 1989). The advent of electricity facilitated further human intervention on a larger scale *via* artificial incubation and brooding, which provided humans with tools to manipulate the photoperiod and thus maintain persistent egg production. The gasoline engine and railroad for transportation allowed for more interactions among geneticists, facilitating exchange of ideas. These, plus the emergence of vaccines and understanding of nutritional requirements for growth and reproduction, allowed for year round production and marketing of broilers. Thus, broiler genetics was becoming a specialty area *per se*.

As stated previously, with the rediscovery of Mendelism at the turn of the 20th century, the chicken, because of traits described earlier, became a model animal for genetic research. This fundamental knowledge led to an understanding for the development and application of breeding programs for meat traits that were quantitatively inherited. Broiler breeders had a fountain for broiler genetics research from publicly supported research as its basis, as well as a range of stocks developed by fanciers, many developed before the rediscovery of Mendelism. These, plus an appreciation for quantitatively inherited traits, genotype-environment interactions, genetic correlations, heterosis, and the concept of resource allocations facilitated development of the broiler *per se*, not as a spin-off from the genetics of egg production. Expansion of mass transportation and development of computer technology contributed to specialized breeding programs that capitalized on a short generation interval with mini-generations. The short generation interval (which is often overlooked), plus a moderate to high heritability for body weight, facilitated reduction in broilers reaching market weights at younger ages, which also improved feed efficiency. These are items that should not be ignored when discussing broiler genetics and improvements in broiler performance during the last 70 years.

Husbandry practices and high energy diets were contributing factors, but they were secondary to the dynamics of selection and crossing of specific male and female lines, i.e., breeding and genetics were the primary contributors (e.g., Havenstein et al., 2003). The financial investment was considerable, and thus it was essential for broiler breeders to have control of their parental lines. Basically, they were utilizing Mendelian genetics per segregation and recombination to protect their investments. Thus, while broiler genetics did not precede the founding of the broiler industry, without the genetic paradigm, the global industry would not be where it is today. Development of sophisticated breeding programs capitalized on the availability of science and technology. As stated previously, during the early phases of commercial broiler breeding, there was reliance on readily available science and technology and a broad gene pool. With a short generation interval, capital investments were necessary and considerable. The result was that only a handful of international groups survived. By producing a 4-way cross, they are able to protect their investments.

That a baby chick could survive for a few days on nourishment from the yolk, coupled with development of the fixed wing aircraft, allowed for global distribution of broiler stocks throughout the world. Broiler production is based on breeding programs (i.e., sound broiler genetics). Its shape is a V, where final product has a narrow base for the source of elite stocks. An analogy is the limited number of sources for long distance aircraft for the international airlines.

Broiler genetics has capitalized on a storehouse of genetic material coupled with science and technology developed over decades. It has allowed for application, which has allowed for an industry to provide an inexpensive meat product derived mainly from plant sources to a global consumer. The basic germplasm and research that allowed for the development of the broiler was derived mainly from public funding with little return to the science *per se* from which the programs were based.

The caveat is that the broiler industry (not unlike some other industries) is dependent on a few multinational groups for their basic product. Their main biological tool is the genetics of the broiler. Their goal is to provide a food product—the broiler—to a growing public. Yes, they should support and conduct fundamental research, but, that is, not their function. The timeline from pedigree to broiler covers several generations and considerable resources. It is important that elite broiler breeding programs rightfully are located at multiple sites. This is essential not only in the event of disease outbreaks, but also, for example, climatic disparities and geopolitical issues. Thus, technological advances in network security, cloud, cybersecurity, redundancy, big data, and business continuity have become ever more relevant to successful broiler genetics.

Globally, an ever emerging human population, with serious climate issues, suggests that there will be numerous challenges in the conversion of plant sources to broiler in the years to come (the production of laboratory meat is not within the realm of this essay). The major genetic changes in broiler breeding (e.g., Havenstein et al., 2003; Siegel, 2014) have been “cherry picked” from the availability of base populations, moderate to high heritability for important traits, and a short generation interval. Credit is given to those who took advantage of these items and realized that broiler breeding should be specific unto itself. Namely, broiler genetics is a subset of genetics *per se* and the broiler is the result of a complex biological system involving the life cycle of its genetic history.

The plateau in broiler genetics will not be for body weight and accompanying positively correlated traits. Body weight is a trait influenced by many genes with small effects (Lillie et al., 2018). It is multifaceted and thus an issue with its genetic variation (as we know from Darwin, the lifeblood of a breeding program is genetic variation) and how to use it. The challenge is from biological and economic constraints of allocation of resources. Because the broiler as the final product is immature when marketed, reproduction cannot be ignored. Broiler breeders have to produce fertile eggs. Biologically, there is competition for mesodermal, endodermal, and ectodermal branches of development. This balancing among resources and allocations are seen in neural and metabolic factors associated with skeletal (e.g., Siegel et al., 2019), muscular (e.g., Petracci et al., 2019), cardiovascular (e.g., Wideman et al., 2013), food consumption (e.g., te Pas et al., 2020), and additional (perhaps unseen) issues. An ironic example is the replacement of plant sources in broiler diets with insects, once considered a pest (van Huis and Gasco, 2023). In this context, not to be dismissed is the coevolution of the microbiome and the hologenome concept (Yang et al., 2017; Zhou et al., 2022a; Zhou et al., 2022b). Such recent discoveries and technological advances provide new tools and challenges for the broiler breeder in the application of broiler genetics.

To address this dynamic for competition for biological resources will require greater interactions, recognizing the sensitivity of proprietary rights and access of information to the scientific community and general public. This interface will not occur “overnight”, because public funding for broiler genetic research has declined. This has contributed to there being just a limited number of public institutions with the capability to train the next-generation of broiler geneticists, i.e., a basic understanding of the interface of the biology of avian species (poultry *per se*) with the technical skills necessary for the application of genetics in broiler breeding. Broiler genetics is the V of broiler breeding. Just as the distance from the primary breeder to the broiler *per se* is great, so is the distance from genotype to phenotype. This biological process is multifaceted, complex, and challenging. Be it broiler breeding or broiler genetics, the “kettle” is far from full. Thus, in concluding this essay, the quote from Eric Hoffer may be appropriate—“The only way to predict the future is to have the power to shape it.”
